# Melatonin Enhances Muscle Development and Suppresses Fat Deposition in Cashmere Goats by Implicating Gut Microbiota and Ameliorating Systemic Antioxidant Status

**DOI:** 10.3390/antiox15010011

**Published:** 2025-12-21

**Authors:** Zhenyu Su, Zibin Zheng, Mulong Lu, Di Han, Jiaxin Qin, Tianzhu Yin, Zhiguo Quan, Shiwei Ding, Liwen He, Wei Zhang

**Affiliations:** 1 State Key Laboratory of Animal Nutrition and Feeding, College of Animal Science and Technology, China Agricultural University, Beijing 100193, China; suzhenyu@cau.edu.cn (Z.S.); zhengzibin@cau.edu.cn (Z.Z.); lml242511@cau.edu.cn (M.L.); handi790302@163.com (D.H.); qinjiaxin@cau.edu.cn (J.Q.); yintianzhu@cau.edu.cn (T.Y.); helw@cau.edu.cn (L.H.); 2 Liaoning Agricultural and Rural Development Service Center, Shenyang 111000, China; 15904206526@163.com (Z.Q.); daweige317@126.com (S.D.)

**Keywords:** melatonin, cashmere goat, muscle development, gut microbiota, antioxidant capacity

## Abstract

Goat meat is widely valued as a healthy option due to its lean nature, yet strategies to further optimize its intrinsic nutritional composition remain a key objective. This study examined the influence of melatonin on muscle development and visceral fat deposition in cashmere goats, focusing on its role in augmenting systemic antioxidant capacity and modifying gut microbiota. Thirty goat kids were randomly assigned to a control or a melatonin-treated (2 mg/kg body weight) group. Melatonin implantation induced a metabolic shift characterized by reduced visceral fat deposition (perirenal, omental, and mesenteric fat; *p* < 0.05) without impacting intramuscular fat. Concurrently, it promoted muscle accretion, as demonstrated by an increase in crude protein content and hypertrophy of muscle fibers in the Longissimus thoracis et lumborum, Gluteus medius, and Biceps femoris muscles (*p* < 0.05). These effects were underpinned by an enhanced systemic antioxidant capacity (elevated CAT, GSH-Px, T-AOC, and reduced MDA; *p* < 0.05), changes in gut microbiota, and a concomitant improvement in gastrointestinal morphology, evidenced by increased rumen papilla length and intestinal villus height. Melatonin enriched beneficial genera (e.g., *Succiniclasticum*, *Butyrivibrio*, *Akkermansia*), which were significantly correlated with reduced adiposity and improved protein deposition. These improvements resulted from the concerted actions of an enhanced systemic antioxidant defense system and a beneficially modulated gut microbial community. This trial observed no effect on intramuscular fat deposition, suggesting that improving intramuscular fat may require a systematic fattening regimen. This study provides a scientific foundation for employing melatonin as a nutritional strategy in goat production to improve meat quality.

## 1. Introduction

Goat meat aligns with modern health trends as a nutritious source of high-quality protein, vitamins, and low cholesterol, making it increasingly valued amid growing demand for wholesome meat driven by rising living standards [1,2]. The cashmere goat is a unique genetic resource in China, primarily distributed across the country’s mountainous regions and highland areas [3]. Meat is the primary product of the cashmere goat industry, alongside cashmere, and constitutes a significant source of income for herders [4]. Current research has largely focused on increasing cashmere production through nutritional interventions, while the systematic improvement of meat quality has received relatively little attention [5,6]. In practical production, the quality of meat often faces numerous challenges, such as excessive body fat leading to inflammation and deterioration in texture caused by oxidative stress [7,8]. Therefore, it is crucial to explore novel strategies that can improve metabolic function and enhance meat quality synergistically, in order to ensure the sustainable development of the cashmere goat industry.

The gastrointestinal tract constitutes a complex ecological niche, comprising host tissues, resident microbial communities, and available dietary substrates [9]. The gut microbiota represents a diverse and resilient consortium of microorganisms, whose structural and compositional integrity is fundamentally connected to host physiological status [10]. It was first observed clinically that gut microbiota can influence muscle metabolism and function through metabolites such as short-chain fatty acids and bile acids [11]. Subsequent studies on livestock and poultry have demonstrated that the addition of exogenous supplements can enhance meat quality and flavour through the gut microbiota [12,13]. Gut microbiota play a pivotal role in regulating body fat deposition, energy metabolism, and promoting muscle synthesis [14,15,16]. Gut microbiota has been demonstrated to be closely associated with meat quality [17].

Melatonin, an endogenous indoleamine, has garnered significant research interest due to its diverse biological activities, which encompass potent antioxidant and anti-inflammatory properties [18,19]. Melatonin participates in a wide range of physiological processes, such as metabolic regulation and skeletal muscle development [20,21]. In livestock production, melatonin is widely utilized for regulating immune responses, promoting gut health, and enhancing meat quality [4,22,23]. Research indicates that melatonin interacts with gut microbiota, with melatonin acting as a dietary supplement to positively affect the homeostasis of animal gut microbiomes [24,25]. Although preliminary studies have demonstrated the positive potential of exogenous melatonin in improving meat quality in ruminants, existing research remains relatively scarce.

Based on previous findings, we hypothesize that melatonin improves meat quality in cashmere goats by promoting muscle development and reducing fat deposition. We further propose that these effects are mediated through the synergistic actions of an enhanced systemic antioxidant capacity and a reshaped gut microbiota, which collectively improve metabolic homeostasis. This study aims to decipher these interconnected mechanisms, thereby providing a scientific foundation for melatonin use as a dietary supplement in sustainable cashmere goat production.

## 2. Materials and Methods

### 2.1. Animal Management

Thirty male half-sibling cashmere goat kids (*Capra hircus*) of the Liaoning breed, at an age of 10 ± 5 days, comprised the experimental cohort. These animals were randomly allocated to either a control or a melatonin-treated group, each containing 15 individuals. The control group exhibited a mean starting weight of 5.17 ± 0.30 kg, while the melatonin group averaged 5.11 ± 0.21 kg. On postnatal days 15, 75, and 135, kids in the melatonin-treated group were administered commercially available, slow-release subcutaneous implants (Kangtai Biotechnology Co., Ltd., Beijing, China) at the base of the left ear. The melatonin-treated group received a dose of melatonin (2 mg/kg live weight) postnatally, as determined by the research team’s preliminary studies [26]. The sustained-release period following melatonin implantation lasted two months.

The eight-month trial (March to November) was conducted at an agricultural facility at a production facility (41°16′ N, 123°12′ E) located in Liaoyang City, within China’s Liaoning Province. All thirty kids were co-housed and managed together throughout the study. They were kept in a pen system comprising an outdoor exercise yard with a compacted earth floor (120 m^2^) and an indoor resting area with slatted floors (120 m^2^), providing a total space allowance of 240 m^2^ for the entire cohort, equivalent to 8 m^2^ per animal. Animals received routine health care, including standard vaccinations, and remained clinically healthy with no disease outbreaks. The basal diet (composition detailed in Table 1) was offered in controlled amounts twice daily at 08:00 and 14:00 h, with fresh water available *ad libitum*.

At eight months of age, five kids per group, with body weights approximating the group mean (32.75 ± 0.71 kg), were selected for slaughter. Prior to slaughter, morphometric assessments were performed on the conscious, standing animals: shoulder height (vertical distance from the scapular summit to the ground), body length (measured from the scapular spine to the pin bone), along with thoracic circumference (taken horizontally just behind the shoulder blade). Following an overnight fast, animals were humanely slaughtered via electrical stunning and exsanguination. All postmortem procedures were conducted on one side of the carcass. Immediately thereafter, venous blood samples were collected by jugular venipuncture. Carcass measurements recorded postmortem included the ribeye area, the GR value (measured as tissue depth at a point 11 cm lateral to the dorsal midline between the 12th and 13th ribs), and backfat thickness (subcutaneous adipose depth at the 12th–13th thoracolumbar junction). The mass of the head, hooves, heart, liver, spleen, lungs, kidneys, testes, and rumen was recorded and subsequently computed for the calculation of respective organ indices. For compositional analysis, entire muscle sections were excised from the Longissimus thoracis et lumborum (LTL) at the thoracolumbar junction, the Gluteus medius (GL) at the central portion of the gluteal region, and the Biceps femoris (BF) from the mid-region of the lateral thigh, and their meat quality traits, crude protein, and ether extract contents were determined. Perirenal, omental, and mesenteric adipose depots were collected and weighed. For microbial analysis, rumen fluid was aseptically collected, and luminal contents were aseptically collected from the terminal ileum, cecum, proximal colon, and rectum. For histological examination, full-thickness tissue sections were collected from the ventral rumen wall, the duodenum (mid-section), the jejunum (mid-section), and the ileum (terminal section), and fixed by immersion in 4% paraformaldehyde. Fixed tissues were processed through paraffin embedding, followed by sectioning and staining with hematoxylin-eosin (H&E).

### 2.2. Carcass Trait Analysis

Thirty minutes postmortem, the hot carcass weight (post-evisceration) was recorded, and the dressing percentage was calculated accordingly. For planimetric assessment of the area, the outline of the LTL cross-section at the thoracolumbar junction was transferred onto acid-fast sulfite paper (Whatman Grade 597; Cytiva, Marlborough, MA, USA). A measuring grid composed of 1 cm squares (Fisher Scientific International, Inc., Hampton, NH, USA) was superimposed on the tracing, and the cross-sectional area was determined by counting complete squares and applying a cardinal-direction rule to partial squares (including upper and left borders, excluding lower and right borders).

### 2.3. Assessment of Meat Quality

Muscle pH readings were obtained using a calibrated Testo 205 pH probe (Testo SE & Co. KGaA, Titisee-Neustadt, Germany) placed within triplicate uniform samples (5 cm × 1 cm × 0.5 cm) obtained from the LTL, BF, and GL muscles [4]. Meat color was characterized on the basis of L* (lightness), a* (redness), and b* (yellowness) values, which were recorded from freshly cut surfaces with a TC-P2A colorimeter (Beijing Aoike Optoelectronic Technology Co., Ltd., Beijing, China) under standardized settings, employing a 5 mm aperture, a D65 light source, and a 10° viewing angle.

Drip loss was determined using freshly excised muscle portions (around 30 ± 2 g), which were prepared for testing within one hour of sampling. A vertical suspension system was employed to preclude compressive contact: each sample was hung from a 2 mm diameter stainless-steel hook inside an airtight polyethylene vessel measuring 15 cm in diameter and 20 cm in height. Samples were stored for 24 h under controlled conditions (2–4 °C, 95% RH). Drip loss percentage was then determined using the standard formula based on weight change.

To prevent evaporative losses during thermal processing, each sample was sealed under vacuum within polyethylene bags (VAC-100; 80 μm thick; 80 μm thick; water vapor permeability: 3 g m^2^/24 h at 25 °C; Beijing Saizhenbo Technology Co., Ltd., Beijing, China). Samples were cooked in a 75 °C water bath for 45 min. Cooking yield was then assessed according to the formula: [(Initial weight − Final weight)/Initial weight] × 100. All assays were performed with three replicates, and the arithmetic mean of these measurements was employed in further statistical evaluations [4].

A texture analyzer (C-LM3, Northeast Agricultural University, Harbin, China) was employed to measure shear force. The measurement was conducted using a shearing blade assembly perpendicular to the orientation of the muscle fibers. This assembly consists of a fixed blade and a pair of movable blades. The fixed blade features a triangular notch with a lower corner radius (R) ≥ 6 mm, and the clearance between the fixed and movable blades on both sides is precisely maintained at 0.3 ± 0.01 mm. This instrument is designed to simulate the shearing action of human mastication, measuring the shear value of muscle and other food samples, with results displayed digitally. The analysis followed established methodology. Water bath was used to heat the samples at 72 °C. The heating proceeded until the internal temperature reached 70 °C and remained constant, indicating thermal equilibrium. Subsequently, the samples were allowed to cool to ambient conditions. Cylindrical samples (1.27 cm in diameter) were then harvested from the muscular tissue by means of a rotating core borer, ensuring the device was oriented parallel to the direction of the muscle fibers. Measurements were taken in triplicate per sample. The highest force value observed as the blade cut through the sample was captured and normalized relative to its cross-sectional area. Values are given as mean ± SD [4].

### 2.4. Measurement of the Chemical Profile of Musclesn

Proximate analysis, utilizing standard AOAC methodologies, was conducted in duplicated experiments to ensure reliability [4]. All results are reported as a percentage of the initial wet sample weight. Moisture content (expressed as a percentage) was assessed by drying samples in an oven at 105 °C until a constant mass was achieved. The value was calculated using the formula: (weight loss during drying/initial wet weight) × 100. The crude protein percentage was quantified through Kjeldahl analysis. Protein content was derived by multiplying the measured nitrogen content by the standard factor of 6.25, with results expressed as a percentage of the original sample’s wet weight. Ether extract (crude fat) content (%) was assessed by Soxhlet extraction and calculated as (weight of fat extracted/wet sample weight) × 100 [27].

### 2.5. Tissue Histology Examination

Tissue samples collected from skeletal muscle, rumen, duodenum, jejunum, and ileum were preserved in 4% paraformaldehyde and then prepared for paraffin embedding. For microscopic evaluation, consecutive tissue sections with a thickness of 5 μm were obtained with a rotary microtome (Leica RM2235, Leica Microsystems GmbH, Wetzlar, Germany) [28]. For microscopic evaluation, the sections were stained with hematoxylin and eosin (H&E). Light microscopic observation and image acquisition were performed using a microscope camera system (Leica ICC50 W; Leica Microsystems). Using ImageJ 1 (National Institutes of Health, Bethesda, MD, USA), the height of ileal villi and the depth of crypts were determined through quantitative morphometric analysis. For the quantification of muscle fibers, digital images of H&E-stained transverse sections were captured under consistent high-power magnification using standardized microscope and camera settings. Muscle fibers were counted manually within multiple, non-overlapping fields of view per sample. Critically, the image acquisition parameters were held constant across all samples, allowing for direct comparison of fiber counts between groups. The data are presented as the mean count of muscle fibers observed per microscopic field for each individual sample [6].

### 2.6. Serum Sample Collection and Biochemical Assessment

Venous blood (5 mL per animal) was drawn aseptically from the jugular vein of each kid. After allowing the blood to clot, the resulting serum was separated and stored in liquid nitrogen for subsequent biochemical analysis. Serum levels of key biomarkers were analyzed using commercial assay kits (Nanjing Jiancheng Bioengineering Institute, Nanjing, China) following the manufacturer’s instructions [29]. The analytes included: (i) antioxidant indices—catalase (CAT), total antioxidant capacity (T-AOC), superoxide dismutase (SOD), glutathione peroxidase (GSH-Px), and malondialdehyde (MDA); (ii) lipid metabolites—triglycerides (TG), total cholesterol (T-CHO), low-density lipoprotein cholesterol (LDL-C), and high-density lipoprotein cholesterol (HDL-C); and (iii) hepatic and renal function markers—blood urea nitrogen (BUN), creatinine (CRE), glutamic-oxaloacetic transaminase (GOT), glutamic-pyruvic transaminase (GPT), and alkaline phosphatase (AKP).

### 2.7. Gastrointestinal Microbiota Analysis

Upon completion of the slaughter procedure, luminal contents from the digestive tract were harvested under aseptic conditions. Following collection, samples were flash-frozen in liquid nitrogen and subsequently maintained at −80 °C for archival storage ahead of microbial composition analysis. Total RNA from the microbial community was isolated employing the AllPrep^®^ PowerFecal^®^ Pro DNA/RNA Kit (QIAGEN GmbH, Hilden, Germany). The purified RNA was then used as a template for cDNA synthesis with SuperScript™ II Reverse Transcriptase (Invitrogen Corporation, Carlsbad, CA, USA). Using primers targeting conserved 16S rRNA regions (341F/805R), one-step PCR was performed, followed by adapter ligation and barcoding. The PCR amplicons were subjected to agarose gel electrophoresis for verification. Separation was performed on a 1.5% gel (in TAE buffer) run at 120 V for 30 min in a Sub-Cell^®^ GT system (Bio-Rad Laboratories, Inc., Hercules, CA, USA) connected to a PowerPac™ Basic power supply. The resulting gel was then imaged with a GelDoc™ XR+ system (Bio-Rad) to confirm product size and specificity. Following electrophoresis, gel fragments containing amplicons of approximately 460 bp were cut out and subjected to purification using the QIAquick Gel Extraction Kit (QIAGEN). Purified amplicons were quantified using Qubit 4.0 (Invitrogen). Equimolar pooling of the purified PCR products was performed. The pooled library was then subjected to paired-end sequencing (250 bp read length) using an Illumina NovaSeq 6000 system [30].

Processing of the raw sequence data involved initial quality control and preprocessing steps. Primer and adapter sequences were trimmed with the aid of cutadapt (v1.9). FLASH software (version 1.2.8) was then used to join the paired-end reads, enforcing a minimum overlap of 10 base pairs and a maximum mismatch allowance of 0.25. Low-quality sequences were filtered using fqtrim (v0.94) with a sliding window of 100 bp: reads with average quality < 20 were truncated, and those shorter than 100 bp or containing >5% ambiguous bases (N) were discarded. To remove chimeras, sequences were processed with Vsearch (version 2.3.4). High-resolution amplicon sequence variants (ASVs) were subsequently inferred through the DADA2 pipeline implemented within the QIIME2 environment, which performs error correction and denoising, and singletons (ASVs with total count = 1 across all samples) were removed. Using the feature-classifier plugin within QIIME2, sequences were taxonomically assigned against the SILVA database (v138) and the NT-16S database (20230718). Confidence thresholds of 0.7 and 90% sequence identity were applied for the two databases, respectively. Alpha diversity was assessed using the following indices calculated in QIIME2: observed ASVs (richness), Chao1 (estimated richness), Shannon (diversity accounting for abundance and evenness), and Simpson (dominance). Good’s coverage was also computed to evaluate sequencing depth. Principal coordinate analysis (PCoA) was employed to visualize beta diversity, which was quantified based on Bray–Curtis distances calculated from ASV abundance data within the QIIME2 pipeline. For biomarker discovery with LEfSe (via the OmicStudio platform; https://www.omicstudio.cn/tool/ (accessed on 17 December 2025)), criteria were set at an LDA score > 4.0 and *p* < 0.05. This step was preceded by differential abundance analysis using Wilcoxon rank-sum tests (*p* < 0.05). This procedure was conducted employing the LEfSe tool established by Segata et al.

### 2.8. Statistical Analyses

All data, including slaughter performance, meat quality parameters, muscle chemical composition, and serum biochemical indices, were subjected to statistical analysis using SPSS version 25.0 (IBM Corp., Armonk, NY, USA). The experimental design was completely randomized, with the treatment group as a fixed effect and each individual animal serving as the experimental unit. All analyses were conducted on the same subset of animals that successfully completed the slaughter procedure, ensuring that data for all measured indicators were derived from identical individuals. Measurements taken from different muscles of the same animal were treated as related observations rather than independent replicates within a group, thereby avoiding pseudoreplication. Between-group comparisons were performed separately for each muscle type and parameter of interest using unpaired two-tailed Student’s *t*-tests after verifying normality (Shapiro–Wilk test) and homogeneity of variances (Levene’s test). Direct statistical comparisons among the three muscle types were not conducted, as the study was designed to evaluate treatment effects rather than to compare muscles per se. Relationships among gut microbiota composition, antioxidant markers, muscle components, and fat deposition were examined using Spearman’s rank correlation analysis. Figures were generated using GraphPad Prism 7 (GraphPad Inc., San Diego, CA, USA). Data are expressed as the mean ± standard deviation (SD). Statistical significance was established at *p* < 0.05.

## 3. Results

### 3.1. Slaughter Performance

Melatonin implantation did not influence preslaughter live weight, carcass weight, dressing percentage, or key morphometric parameters (withers height, body length, chest circumference, cannon bone circumference, and GR value) in Liaoning cashmere goats (*p* > 0.05), while reducing head weight (*p* < 0.05). Furthermore, melatonin implantation reduced the weights of perirenal, omental, and mesenteric fat (*p* < 0.05), thereby alleviating visceral fat accumulation (Table 2).

The administration of melatonin did not alter the absolute mass of the heart, liver, spleen, lungs, or kidneys (*p* > 0.05). However, it increased the organ index of the lungs (*p* < 0.05, Table 3).

### 3.2. Muscle Characteristics and Meat Quality

**Figure 1:**
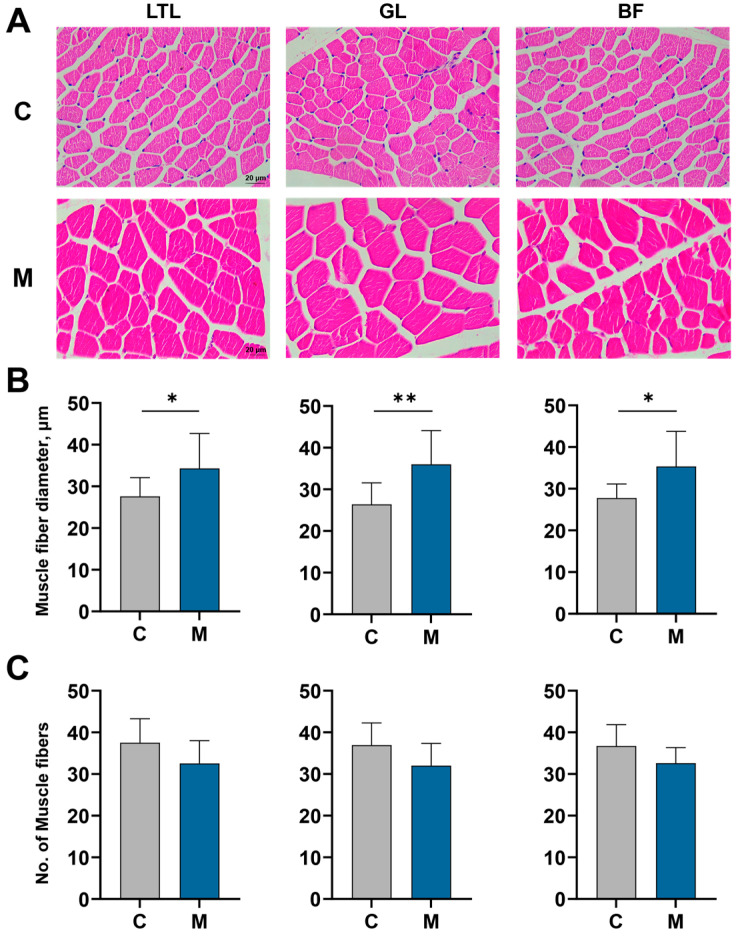
Histological and morphometrical properties of the Longissimus thoracis et lumborum (LTL), Gluteus (GL), and Biceps femoris (BF) muscles in control (C) versus melatonin-implanted (M) cohorts at slaughter. (**A**) Typical hematoxylin and eosin (H&E)-stained images (Scale bar corresponds to 20 µm), (**B**) Measurement of muscle fiber diameter, (**C**) Comparison of relative muscle fiber counts across the three muscle types. Results are expressed as mean ± SD. Significance is indicated with asterisks (* *p* < 0.05, ** *p* < 0.01).

Melatonin exerted distinct effects across muscle anatomical locations (Table 4). In the longissimus thoracis et lumborum (LTL), treatment elevated 24 h post-mortem pH (*p* < 0.05), while concurrently enhancing crude protein content (*p* < 0.05). Within the gluteus medius (GL), melatonin diminished drip loss and increased crude protein content (*p* < 0.05). In the biceps femoris (BF), melatonin implantation reduced drip loss (*p* < 0.05), while elevating crude protein levels (*p* < 0.05, Table 4). Histological examination via H&E staining demonstrated that melatonin increased muscle fiber diameter across all three muscles (*p* < 0.05), but did not alter relative muscle fiber number (*p* > 0.05, Figure 1A–C).

### 3.3. Blood Biochemical Parameters

**Figure 2:**
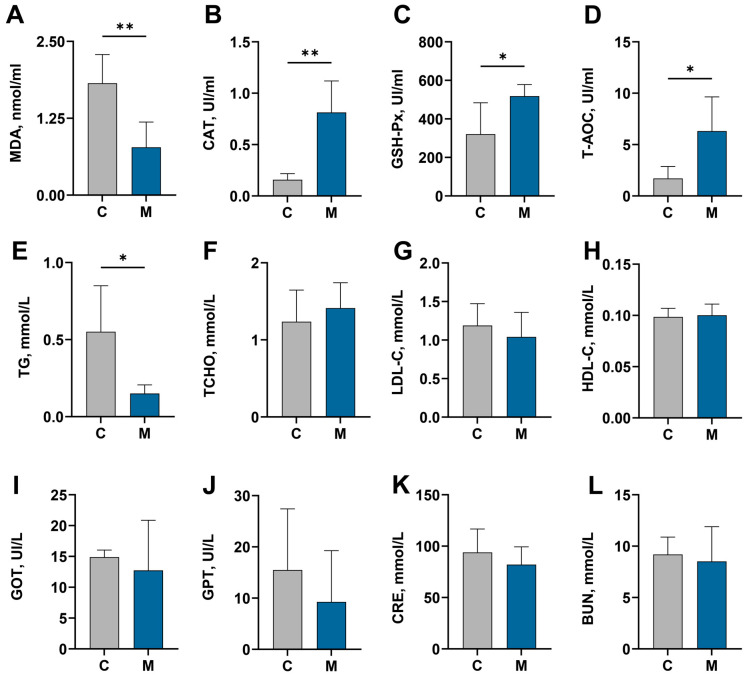
Serum biochemical indices for the control (C) and melatonin-treated (M) groups. (**A**–**D**) Antioxidant indices: malondialdehyde (MDA), catalase (CAT), total antioxidant glutathione peroxidase (GSH-Px) and capacity (T-AOC). (**E**–**H**) Lipid metabolism markers: triglyceride (TG), total cholesterol (TCHO), low-density lipoprotein cholesterol (LDL-C), and high-density lipoprotein cholesterol (HDL-C). (**I**–**L**) Liver and kidney function parameters: glutamic oxaloacetic transaminase (GOT), glutamic pyruvic transaminase (GPT), creatinine (CRE), and blood urea nitrogen (BUN). Results are expressed as mean ± SD. Significance is indicated with asterisks (* *p* < 0.05, ** *p* < 0.01).

The assessment of oxidative stress severity can be achieved by quantifying the levels of catalase (CAT), glutathione peroxidase (GSH-Px), malondialdehyde (MDA), and total antioxidant capacity (T-AOC) [31]. Experimental results indicate that melatonin administration elevated CAT and GSH-Px activity alongside T-AOC levels in cashmere goats (*p* < 0.05), while markedly reducing MDA levels (*p* < 0.05, Figure 2A–D), thereby enhancing the animals’ antioxidant capacity. Lipid metabolism indicators showed that melatonin implantation reduced blood TG levels (*p* < 0.05, Figure 2E), but did not alter TCHO, LDL-C, or HDL-C levels (*p* > 0.05, Figure 2F–H). Furthermore, melatonin administration was associated with decreased serum activities of the hepatic enzymes GOT, GPT, and AKP in cashmere goats. However, these values were similar to those of the control group (*p* > 0.05; Figure 2I–L).

### 3.4. Histological Structure of the Digestive Tract and Composition of the Gut Microbiota

**Figure 3:**
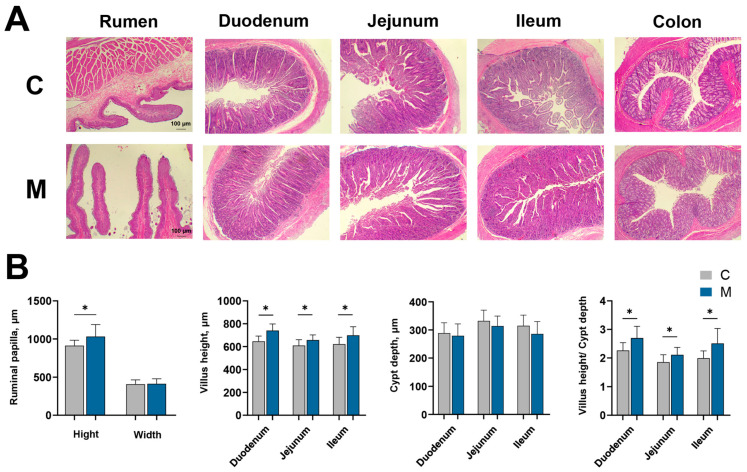
Digestive tract histology and microbial community profiles in control (C) and melatonin-treated (M) upon slaughter. (**A**) Representative images of H&E-stained tissues from the rumen, duodenum, jejunum, ileum, and colon (Scale bar corresponds to 100 µm), (**B**) Quantification of villus height and crypt depth. Results are expressed as mean ± SD. Significance is indicated with asterisks (* *p* < 0.05).

Histomorphometric measurements revealed that melatonin treatment had a positive effect on the architecture of the rumen and intestines (Figure 3A). Histomorphometric evaluation demonstrated an enhancement of rumen papilla length following melatonin implantation (*p* < 0.05), with no effect on width. Furthermore, villus height in the duodenum, jejunum, and ileum was elevated in melatonin-administered goats compared to controls (*p* < 0.05). Nevertheless, crypt depth did not differ between the melatonin-treated and control groups in any of the intestinal segments examined (*p* > 0.05, Figure 3B).

**Figure 4:**
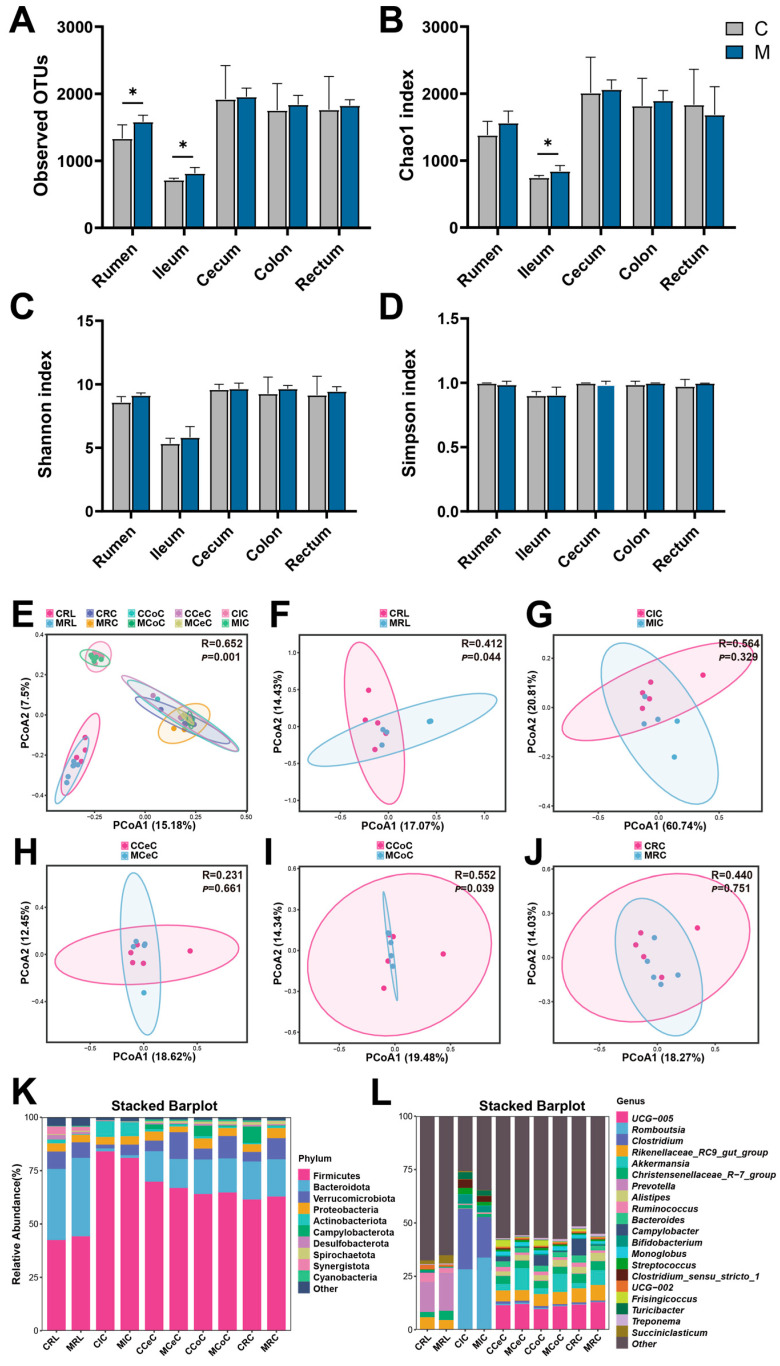
Microbial community diversity and taxonomic composition in Liaoning cashmere goats. (**A**–**D**) Assessment of microbial alpha diversity (OTU, Chao1, Shannon, Simpson) across distinct gastrointestinal tract segments: rumen, ileum, cecum, colon, and rectum. (**E**–**J**) Bray–Curtis distance-based PCoA illustrating the β-diversity of bacterial communities in digesta samples collected from the rumen (**F**), ileum (**G**), cecum (**H**), colon (**I**), and rectum (**J**). (**K**,**L**) Composition of the microbiota at the phylum (**K**) and genus (**L**) taxonomic levels. Results are expressed as mean ± SD. Significance is indicated with asterisks (* *p* < 0.05).

Analysis of α-diversity in gastrointestinal microbial communities showed that melatonin treatment had segment-specific effects on the microbiota of the digestive tract in male cashmere goats. In terms of community richness, melatonin treatment increased the OUT index in both the rumen and the ileum (*p* < 0.05, Figure 4A). The Chao1 index for ileal microbiota also increased in the melatonin group (*p* < 0.05, Figure 4B). Nonetheless, neither the Shannon nor the Simpson index showed intergroup differences (*p* > 0.05, Figure 4C,D). Analysis of Bray–Curtis distances through PCoA indicated structural variation in the microbiota associated with distinct gastrointestinal tract segments. PERMANOVA analysis showed that melatonin treatment altered microbial community structures in the rumen and colon (*p* < 0.05, Figure 4E,H), but not in the ileum, caecum or rectum (*p* > 0.05, Figure 4F,G,I).

**Figure 5:**
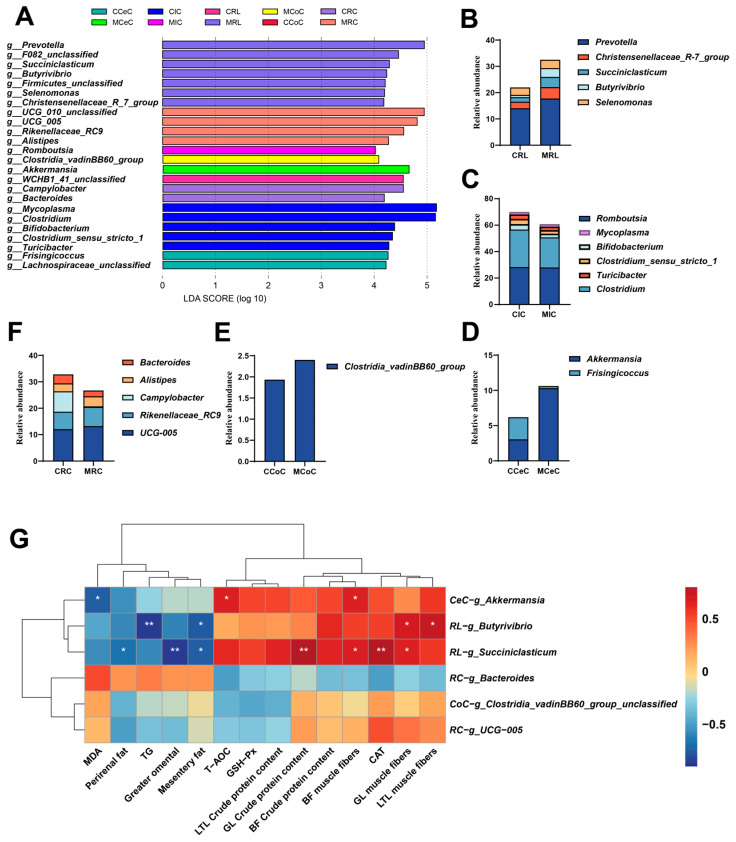
Microbial taxa and treatment-associated signatures in Liaoning cashmere goats, alongside a correlational assessment between phenotypic traits and microbial genera using Spearman’s method. (**A**–**F**) LEfSe analysis revealed bacterial genera exhibiting differential abundance (Linear Discriminant Analysis LDA score > 4.0). across distinct gastrointestinal regions, including the rumen (**B**), ileum (**C**), caecum (**D**), colon (**E**), and rectal digesta (**F**). (**G**) Heatmap of Spearman correlations between host phenotypes and bacterial genera in control and melatonin-administered. Statistical significance: * *p* < 0.05, ** *p* < 0.01.

Dominance in the gut ecosystem of cashmere goats was observed for the bacterial phyla Firmicutes, Bacteroidetes, Verrucomicrobia, Proteobacteria, and Actinobacteria (Figure 4J). At the genus level, the core genera were primarily *UCG-005*, *Romboutsia*, *Clostridium*, *Rikenellaceae_RC9* and *Akkermansia* (Figure 4K). Based on LDA > 4.0 analysis using LEfSe, this study identified 24 microbial taxa that exhibited intergroup differences (Figure 5A–F). Specifically, melatonin treatment enriched the relative abundance of *Succiniclasticum* and *Butyrivibrio* in the rumen, and of *Akkermansia* in the cecum (*p* < 0.05; Figure 4B,F). Concurrently, the abundance of *Clostridia_vadinBB60_group* in the colon and of *UCG-005* in the rectum increased (*p* < 0.05 Figure 4E,D), conversely, melatonin treatment reduced the relative abundance of *Bacteroides* residing in the rectum (*p* < 0.05, Figure 4D).

To investigate the intrinsic relationship between melatonin-regulated, differential microbiota and key host phenotypes, we conducted Spearman’s rank correlation analyses between significantly altered bacterial genera and antioxidant indicators, lipid metabolism parameters, and meat quality traits (Figure 5G). The results indicated that *Akkermansia* exhibited a positive correlation with LM muscle fibre number and total antioxidant capacity (*p* < 0.05), and exhibited an inverse relationship with MDA content (*p* < 0.05). *Bacteroidetes* showed a positive association with muscle fiber counts in both the TL and GL muscles (*p* < 0.05), while demonstrating an inverse relationship with mesenteric fat (*p* < 0.05). *Succiniclasticum* exhibited positive correlations with muscle fibre count, CAT activity, and crude protein content in GL and BF (*p* < 0.05), and negative correlations with mesenteric fat, omental fat, and perirenal fat (*p* < 0.05).

## 4. Discussion

Although the influence of exogenous melatonin on carcass characteristics has been extensively studied across various species, conclusive findings remain elusive [32]. Earlier investigations have reported that melatonin inclusion in broiler diets does not significantly influence overall live weight or dressed carcass weight at slaughter [33]. Similar experimental results have been obtained in studies on pigs and cattle [34,35]. Extending our investigation from females to males [6], the present study in male Inner Mongolian cashmere goats demonstrates that exogenous melatonin similarly has a limited impact on overall carcass characteristics. However, further analysis revealed that melatonin treatment significantly reduced the deposition of perirenal fat, omental fat, and mesenteric fat. This key anti-lipogenic effect aligns with our previous observations in female goats [6], suggesting a conserved role of melatonin in modulating visceral fat metabolism across sexes in this species. Nonetheless, subtle differences were noted between sexes, highlighting the importance of considering sex as a biological variable in nutritional interventions. Our findings in males consolidate the potential of melatonin as a metabolic regulator targeting fat deposition, while underscoring the need for further research to fully elucidate its sex-specific mechanisms.

The positive effects of this metabolic modulation are clearly evident in the quality of the meat across multiple muscle sites. Lumborum (LTL), gluteus (GL) and biceps femoris (BF) are the three key muscle sections of the cashmere goat [6]. The positive effects of this metabolic modulation are clearly evident in the quality of the meat across multiple muscle sites. Lumborum (LTL), gluteus (GL) and biceps femoris (BF) are the three key muscle sections of the cashmere goat [6]. Melatonin administration led to a pronounced reduction in drip loss in both the GL and BF muscles. Analysis of muscle histology further demonstrated a significant increase in the relative abundance of muscle fibers in the LTL, GL, and BF regions. Additionally, the LTL, GL, and BF muscles exhibited a marked increase in crude protein content. The crude protein content of muscle is widely recognized as a critical indicator of meat’s nutritional value, due to its direct correlation with whole-body protein deposition [36,37]. Our findings that melatonin supplementation enhanced muscle protein accretion and water retention in cashmere goats are consistent with the broader role of melatonin in promoting muscle development and improving meat quality attributes, as highlighted in a recent comprehensive review [38]. These results highlight the observed effects of melatonin on improving two key meat quality parameters: enhanced water-holding capacity (reduced drip loss) and increased protein content in muscle tissue. This concurrent improvement in both physical and nutritional attributes suggest a multifaceted role for melatonin in muscle metabolism. In contrast to the common focus on intramuscular fat in meat quality studies, our findings from non-fattening goats underscore melatonin’s potency in improving muscle attributes. Studies demonstrate that melatonin influences lipid homeostasis and facilitates the growth and specialization of adipocytes within muscle tissue [39,40]. While some research also notes melatonin’s influence on fat deposition patterns [38], our study specifically reveals its significant effect on reducing visceral fat depots (perirenal, omental, and mesenteric) in goats, a nuance not extensively covered in prior summaries. In this experiment, the stable intramuscular fat content may be linked to the absence of a fattening dietary regime. Therefore, investigating the effects of melatonin in finished goats represents a logical next step.

The freshness and quality of meat are greatly affected by oxidation [41]. Antioxidant capacity is positively correlated with a healthy body and meat quality [42,43]. Within the body, antioxidant enzymes serve as a cornerstone for maintaining redox equilibrium and safeguarding organ integrity [44]. Augmenting their activity thus constitutes a potent means of ameliorating oxidative stress and decelerating oxidative processes [45]. Melatonin and its metabolites have a powerful antioxidant effect, as they scavenge free radicals and enhance the activity of antioxidant enzymes [46]. This alleviates oxidative stress in the body [47]. Prior investigations by our group have shown that melatonin supplementation in cashmere goats augments systemic antioxidant defenses and mitigates markers of oxidative stress [6,48]. These observations align with the outcomes documented in the current investigation. Given that oxidative stress is a major contributor to muscle protein oxidation and quality deterioration, this antioxidant mechanism provides critical evidence explaining the observed meat quality improvements [7]. Furthermore, excessive fat deposition adversely impacts meat quality and safety, and may promote systemic lipid metabolism disorders, thereby increasing the risk of various secondary diseases [49,50]. Furthermore, research indicates that oxidative stress exacerbates lipid metabolism abnormalities [51]. Having confirmed melatonin’s role in alleviating oxidative stress at the systemic level (as indicated by serum antioxidant indices), we extended our analysis to lipid metabolism. The significant reduction in serum triglyceride (TG) levels coincided with the observed phenotype of diminished visceral fat deposition. This correlation suggests that melatonin mitigates abnormal lipid accumulation by modulating fundamental processes of lipid mobilization and utilization.

The results of previous studies have shown that the digestive tract microbial community of ruminants, particularly in the rumen and cecum, plays a crucial role in their fat deposition and antioxidant processes [52,53]. Zhang et al. [52] noted that specific microorganisms in the rumen, such as *Bifidobacterium*, *Butyrivibrio*, and *Prevotellaceae UCG-003*, were significantly associated with fat deposition phenotypes, and that these microorganisms regulate energy utilization and fat accumulation in the host through metabolic pathways. Chen et al. [53], on the other hand, showed that ruminant cecum *Lachnospiraceae_NK3A20_group* can regulate organismal fat deposition through the VFAs-Serum triglyceride metabolic pathway. Conversely, evidence from ruminant studies confirms that melatonin exerts notable effects on the structure and function of the gastrointestinal microbiome. Guo et al. [54] indicated that melatonin was able to restore the balance of the intestinal flora in male dairy goats and to reduce the over-synthesis of arachidonic acid under conditions of heat stress, thus improving the function of spermatogenesis. Previous investigations in female cashmere goats have indicated that melatonin treatment modifies gut microbiota composition by elevating α-diversity and promoting the abundance of certain beneficial bacterial genera [6]. In the current investigation utilizing male cashmere goats, the number of observed OTUs in both ruminal and ileal microbiota was notably greater in the melatonin-treated (M) group relative to the control (C) group. This finding suggests that melatonin administration enhanced the richness of microbial communities residing in the rumen and ileum. Meanwhile, the significant increase in the Chao1 index of ileal microorganisms suggested that melatonin supplementation might enable more rare microbial taxa to be detected, resulting in a more abundant community species pool. In β-diversity analysis, the microorganisms in various intestinal segments were clearly separated, whereas the separation of microorganisms in the cecum, colon, and rectum was not obvious. Meanwhile, the differences in microbial community structure of ruminal fluid and colon between Groups M and C indicated that melatonin supplementation might have affected the digestion and absorption of nutrients in these two parts.

Combining LEFSE analysis and nonparametric tests, we found that the genera *Succiniclasticum* and *Butyrivibrio* were significantly increased in the rumen, *Akkermansia* in the cecum, *Clostridia_vadinBB60_group* in the colon, *UCG-005* in the rectum, while *Bacteroides* in the rectum was significantly decreased. We hypothesize that melatonin forms a health maintenance network covering the entire digestive tract through “SCFA metabolic chain connection and barrier-anti-inflammatory synergy”. In the rumen, the genera *Succiniclasticum* and *Butyrivibrio* are both important taxa, involved in the conversion of succinic acid to propionic acid and cellulose degradation to produce butyric acid, respectively [55,56]. Among them, propionic acid serves as a key energy source for ruminal epithelial cells and the whole organism, while butyric acid promotes the proliferation of ruminal epithelial cells and enhances tight junctions [55,56]. The significant increase in their abundances may improve rumen fermentation efficiency and protect the ruminal epithelium. In the correlation analysis, we also found that these two genera were significantly associated with multiple phenotypic indicators. In addition to being significantly negatively correlated with mesenteric fat, they were extremely significantly negatively correlated with serum TG and omental fat, respectively. It is speculated that butyric acid and propionic acid produced by these two genera can reduce adipose tissue accumulation and blood lipid levels by inhibiting fatty acid synthase and promoting lipolysis [57]. Extending the analysis to male goats, this experiment revealed a novel and significant negative correlation between the genus *Succiniclasticum* and perirenal fat deposition. Furthermore, the two rumen genera mentioned above were also positively correlated with crude protein content and muscle fibre in meat. A proposed mechanism suggests that SCFAs such as acetate and propionate contribute to the promotion of muscle protein synthesis by initiating the mTOR signaling pathway, while improving intestinal absorption function to provide sufficient amino acids for muscle growth [58]. The cecum is the main site of hindgut fermentation in ruminants, responsible for the further decomposition of cellulose and other complex carbohydrates undigested by the rumen [59]. The microbial community in the cecum converts these substances into volatile fatty acids through fermentation [59]. Among them, *Akkermansia* is an important microorganism that regulates protein synthesis and metabolism, promotes the biosynthesis of fats and lipids, and affects muscle fat content [60]. In female goats, we also found a significant negative correlation between this genus and MDA, as well as a significant positive correlation with TAC [6]. The significant increase in *Akkermansia* in this experiment indicates that melatonin may regulate intramuscular fat deposition through cecal microorganisms. The cecum and colon are the primary sites of hindgut fermentation [61]. Especially when rumen fermentation is incomplete, hindgut fermentation can further decompose cellulose and other indigestible plant components, producing volatile fatty acids to further provide energy for ruminants [59]. Research indicates that the genus *UCG-005* in the rectum may be involved in carbohydrate metabolism and energy acquisition, providing energy for the host by decomposing complex polysaccharides [62]. In addition, *UCG-005* may also participate in nitrogen metabolism, helping the host utilize nitrogen sources in the feed more efficiently [62]. Thus, our findings demonstrate that melatonin enhances meat quality in non-fattened Cashmere goats by simultaneously suppressing fat deposition and promoting protein accretion. These improvements result from the concerted actions of an improved systemic antioxidant capacity and a beneficially restructured gut microbiota.

## 5. Conclusions

Melatonin implantation in male cashmere goats significantly reduces visceral fat deposition without compromising overall growth and carcass performance, while in-creased protein content and enhanced water-holding capacity. This effect is closely associated with an enhanced systemic antioxidant capacity and improved lipid metabolism. In-depth investigations reveal that melatonin enriches key metabolically regulatory bacterial genera—such as *Succiniclasticum*, *Butyrivibrio*, and *Akkermansia*—within the gastrointestinal tract, thereby establishing a more beneficial microbial ecosystem. This research provides a theoretical basis for developing melatonin as a nutritional strategy to improve the meat quality of ruminants. The key next step is to validate the causal role of the identified microbiota in mediating these metabolic benefits, and to determine the strategy’s efficacy in industry-relevant high-energy finishing systems.

## Figures and Tables

**Table 1:** Formulation and nutritional composition of the experimental diet (DM basis).

Ingredient	(%)	Nutrient Levels ^2^	
Alfalfa	35.00	ME, (MJ/Kg)	9.69
Peanut straw	35.00	DM, (%)	89.93
Corn	13.08	EE, (%)	3.27
Extruded Corn	4.62	CP, (%)	15.24
Soybean Meal	6.40	NDF, (%)	50.30
Corn Gluten Meal	1.50	ADF, (%)	36.23
Extruded Full-fat Soybean	2.00	Ca, (%)	1.24
Limestone	0.45	P, (%)	0.46
Salt	0.21		
Premix ^1^	0.45		
Molasses	0.90		
Monocalcium Phosphate	0.15		
Ammonium Chloride	0.15		
Choline Chloride	0.03		
Tannic Acid	0.06		
Total	100		

^1^ Premix: FeSO_4_·7H_2_O 170 g/kg; CuSO_4_·5H_2_O 70 g/kg; MnSO_4_·5H_2_O 290 g/kg; ZnSO_4_·7H_2_O 240 g/kg; CoCl_2_·6H_2_O 510 mg/kg; KI 220 mg/kg; Na_2_SeO_3_ 130 mg/kg; VA1, 620,000 IU/kg; VD3 324,000 IU/kg; VE 540 IU/kg; VK3 150 mg/kg; VB1 60 mg/kg; VB2 450 mg/kg; VB12 0.9 mg/kg. ^2^ All nutrient values were obtained by direct analytical measurement, with the exception of metabolizable energy (ME), which was derived by calculation. ME, metabolizable energy; DM, dry matter; CP, crude protein; EE, ether extract; ADF, acid detergent fiber; NDF, neutral detergent fiber.

**Table 2:** Impact of Melatonin Administration on Slaughter Traits.

Item	CON	MT	*p*-Value
Slaughter weight (kg)	33.44 ± 0.45	32.05 ± 1.50	0.448
Carcass weight (kg)	14.70 ± 1.24	14.19 ± 0.84	0.771
Carcass yield (%)	43.83 ± 3.32	44.20 ± 1.21	0.926
Height at withers (cm)	54.05 ± 1.02	54.82 ± 0.96	0.894
Length of body (cm)	54.78 ± 1.22	57.90 ± 1.58	0.225
Circumference of chest (cm)	9.60 ± 0.35	9.58 ± 0.15	0.961
Head weight (kg)	2.36 ± 0.03 ^b^	2.60 ± 0.07 ^a^	0.023
Hoof weight (kg)	0.71 ± 0.09	0.94 ± 0.03	0.071
GR (cm)	3.00 ± 0.21	2.95 ± 0.70	0.975
Rib-eye area (cm^2^)	16.11 ± 1.57	15.40 ± 1.35	0.763
Perirenal fat (g)	274.88 ± 18.91 ^b^	170.42 ± 23.87 ^a^	0.015
Greater omental (g)	655.94 ± 32.52 ^b^	323.48 ± 32.42 ^a^	0.001
Mesentery fat (g)	346.40 ± 24.96 ^b^	196.87 ± 8.71 ^a^	0.013

CON, Control group; MT, Melatonin group. GR: tissue depth at the 12th–13th intercostal space, 11 cm lateral from the dorsal midline. Values are reported as means ± standard deviation (SD), with a sample size of *n* = 5 per group. Differences between groups were evaluated using a two-tailed Student’s *t*-test for independent samples, using *p* < 0.05 as the significance threshold. Significant differences between groups are indicated by distinct lowercase superscript letters.

**Table 3:** Impact of Melatonin on the Visceral Organ Characteristics.

Item		CON	MT	*p*-Value
Weight (g)	Heart	84.64 ± 8.97	95.56 ± 5.83	0.388
	Liver	469.98 ± 39.16	536.24 ± 19.57	0.213
	Spleen	28.42 ± 3.11	35.44 ± 2.64	0.162
	Lung	271.50 ± 19.94	335.58 ± 26.55	0.123
	Kidney	79.36 ± 6.15	88.80 ± 9.74	0.363
Index (%)	Heart	0.25 ± 0.03	0.30 ± 0.01	0.255
	Liver	1.41 ± 0.13	1.68 ± 0.07	0.099
	Spleen	0.09 ± 0.02	0.11 ± 0.02	0.650
	Lung	0.81 ± 0.08 ^b^	1.04 ± 0.06 ^a^	0.041
	Kidney	0.23 ± 0.02	0.28 ± 0.03	0.260
Testes (g)		183.54 ± 17.25	183.14 ± 2.66	0.984
Rumen weight (kg)		3.16 ± 0.75	4.20 ± 0.91	0.454
Net rumen (kg)		0.66 ± 0.05	0.91 ± 0.17	0.222
Rumen pH		6.60 ± 0.39	6.68 ± 0.14	0.870

CON, Control group; MT, Melatonin group. Values are reported as means ± standard deviation (SD), with a sample size of *n* = 5 per group. Differences between groups were evaluated using a two-tailed Student’s *t*-test for independent samples, using *p* < 0.05 as the significance threshold. Significant differences between groups are indicated by distinct lowercase superscript letters.

**Table 4:** Influence of Melatonin on Meat Quality Parameters.

Item		CON	MT	*p*-Value
LTL	pH_45_min	6.42 ± 0.26	6.54 ± 0.27	0.529
	pH_24_h	5.46 ± 0.07	6.16 ± 0.39	0.017
	L*	36.50 ± 0.73	37.12 ± 2.08	0.586
	a*	17.13 ± 1.60	17.27 ± 0.62	0.826
	b*	4.01 ± 0.36	4.13 ± 0.46	0.670
	Drip loss (%)	7.95 ± 3.72	7.26 ± 3.15	0.786
	Cooking loss (%)	50.54 ± 4.85	43.53 ± 6.68	0.251
	Shear force (kgf)	11.26 ± 0.87	13.15 ± 1.98	0.118
	Moisture content (%)	71.02 ± 0.01	71.15 ± 0.01	0.790
	Crude protein content (%)	26.19 ± 0.34 ^b^	27.18 ± 0.38 ^a^	0.032
	Ether extract content (%)	2.03 ± 0.19	1.73 ± 0.25	0.357
GL	pH_45_min	6.40 ± 0.26	6.73 ± 0.23	0.094
	pH_24_h	5.73 ± 0.22	5.78 ± 0.23	0.750
	L*	37.08 ± 1.60	38.17 ± 2.23	0.452
	a*	19.05 ± 1.72	18.50 ± 0.99	0.595
	b*	4.72 ± 0.34	4.89 ± 0.54	0.596
	Drip loss (%)	8.31 ± 3.26 ^b^	3.41 ± 1.01 ^a^	0.021
	Cooking loss (%)	49.61 ± 4.16	50.23 ± 3.67	0.827
	Shear force (kgf)	9.72 ± 3.34	12.88 ± 2.00	0.144
	Moisture content (%)	70.68 ± 0.86	67.62 ± 2.06	0.235
	Crude protein content (%)	24.29 ± 0.66 ^b^	28.39 ± 1.52 ^a^	0.038
	Ether extract content (%)	2.03 ± 0.19	1.73 ± 0.25	0.658
BF	pH 45 min	6.39 ± 0.15	6.76 ± 0.42	0.137
	pH 24 h	5.79 ± 0.20	5.70 ± 0.15	0.483
	L*	37.60 ± 2.06	37.00 ± 1.36	0.643
	a*	17.40 ± 1.70	16.77 ± 0.73	0.514
	b*	4.39 ± 0.35	4.26 ± 0.37	0.604
	Drip loss (%)	9.61 ± 2.72 ^b^	5.44 ± 2.09 ^a^	0.041
	Cooking loss (%)	50.41 ± 4.22	50.88 ± 4.68	0.884
	Shear force (kgf)	12.15 ± 1.86	12.72 ± 0.77	0.583
	Moisture content (%)	70.97 ± 0.30	70.06 ± 0.42	0.113
	Crude protein content (%)	25.56 ± 0.13 ^b^	27.22 ± 0.21 ^a^	0.004
	Ether extract content (%)	2.13 ± 0.20	2.49 ± 0.30	0.335

CON, Control group; MT, Melatonin group. Values are reported as means ± standard deviation (SD), with a sample size of *n* = 5 per group. Differences between groups were evaluated using a two-tailed Student’s *t*-test for independent samples, using *p* < 0.05 as the significance threshold. LTL, longissimus thoracis et lumborum. GL, Gluteus. BF, biceps femoris. Significant differences between groups are indicated by distinct lowercase superscript letters.

## Data Availability

All raw data and sequencing information can be requested by contacting the corresponding author Wei Zhang (wzhang@cau.edu.cn).
